# Auscultatory versus oscillometric blood pressure measurement in patients with atrial fibrillation and arterial hypertension

**DOI:** 10.1186/s12872-017-0521-6

**Published:** 2017-03-23

**Authors:** Aistėja Šelmytė–Besusparė, Jūratė Barysienė, Dovilė Petrikonytė, Audrius Aidietis, Germanas Marinskis, Aleksandras Laucevičius

**Affiliations:** 1 Centre of Cardiology and Angiology, Vilnius University Hospital Santariskiu Clinics, 2 Santariškių St., LT-08661 Vilnius, Lithuania; 2 Clinic of Cardiovascular Diseases, Faculty of Medicine, Vilnius University, 21 M. K. Čiurlionio St., LT-03101 Vilnius, Lithuania

**Keywords:** Atrial fibrillation, Arterial hypertension, Blood pressure monitoring, Oscillometry, Auscultatory method

## Abstract

**Background:**

The aim of our study was to investigate the reliability of automated oscillometric blood pressure (BP) monitoring in the presence and absence of atrial fibrillation (AF) in hypertensive patients.

**Methods:**

BP was measured and compared in 71 randomly selected patients with AF and arterial hypertension diagnosis, 4 times each by auscultatory and oscillometric (Microlife BP A6 PC with AF detection system) methods.

**Results:**

Study included 71 patients: 36 males (mean age 67.4 years) and 35 females (70.2 years). At the time of BP measuring procedure, 36 patients were in sinus rhythm (SR) and 35 in AF. In SR patients mean systolic blood pressure (SBP) was 132 ± 17.9 mmHg with auscultatory method (AM), 137.4 ± 19.4 mmHg with oscillometric method (OM); mean diastolic BP was 77.1 ± 10.9 mmHg (AM), 78.5 ± 12.2 mmHg (OM), in AF patients mean SBP was 127.5 ± 15.1 mmHg (AM), 133.6 ± 17.4 mmHg (OM); mean diastolic BP was 81.4 ± 9.9 mmHg (AM), 83.5 ± 11.8 mmHg (OM), *p* = 0.037. The averages of differences for SBP and DBP in sinus rhythm group were (−5.3 mmHg (95% limits of agreement −27.2 – 16.6)) and (−1.4 mmHg (95% limits of agreement −12.8 – 10.0)), respectively. In patients with AF the averages of differences for SBP and DBP were (−6.1 mmHg (95% limits of agreement −23.9 – 11.7)) and (−2.1 mmHg (95% limits of agreement −12.9 – 8.7)), respectively.

**Conclusions:**

The oscillometric device validated for patients with AF on average gives 5.3 mmHg higher systolic BP values for patients with SR and 6.3 mmHg higher BP values for patients with AF. However, the limits of agreement between two methods reveal wide range of random error rates which is a questionable topic in clinical practice, as it could possibly affect the treatment of arterial hypertension in patients with AF.

**Electronic supplementary material:**

The online version of this article (doi:10.1186/s12872-017-0521-6) contains supplementary material, which is available to authorized users.

## Background

Atrial fibrillation (AF) is the most common sustained arrhythmia [1–3] with the prevalence of 1–2% in general population [4, 5] and it is associated with increased morbidity, mortality and rising usage of health care resources [2]. Arterial hypertension (AH) is one of the most common aethiological factors for AF [6, 7] increasing the risk of AF for both males and females 1.5 and 1.4 times, respectively [8]. Therefore, early diagnosis and effective treatment of AH are essential for patients with AF [7, 9]. Hypertension with concomitant AF increase the risk of a stroke and require regular measuring and blood pressure (BP) control. This may be reached by self–monitoring of BP at home, which is more important than measuring BP at a clinic [9]. Moreover, measuring BP at home may ensure more precise treatment of AH and it may also help to diagnose AH early [10]. In the clinical setting BP is usually measured using manual or automatic devices [11]. Oscillometric method has markedly simplified self–monitoring of BP and it may be a better choice for measuring BP at home [12]. Although oscillometric BP–measuring devices are becoming widespread, they may be considered unreliable for the patients with AF [13] because the devices measure BP from a smooth profile of successive pressure waves [14] and because of high variability of the heart rate and stroke volume during arrhythmia [15]. Furthermore, most automatic BP measuring devices are validated and calibrated only for patients with sinus rhythm (SR) and even manufacturers recommend caution when the devices are used for patients with arrhythmias [12]. On the other hand, nowadays more and more special oscillometric BP devices with AF detectors are used in clinical practice [16].

Previous studies [11, 17] have shown that oscillometric devices perform satisfactorily in AF, if repeated measurements are performed. Despite that, recent systematic review and meta–analysis [18] demonstrated that there is limited evidence in studies that validated the automated BP devices in AF. Meta–analysis concluded that oscillometric devices may be suitable for measuring systolic, but not for diastolic BP and may be appropriate for measuring BP at home, but not for office measurement.

Recent study by Pagonas et al. [12] compared the BP measured by oscillometric device with invasively assessed BP and concluded that AF does not significantly decrease the accuracy of oscillometry after 3 consecutive measurements. However, one of their study’s limitations was that their oscillometric BP measuring devices were not intended for patients with AF.

## Methods

### The aim of the study

The aim of our study was to investigate the reliability of automated oscillometric BP monitoring in the presence and absence of AF in hypertensive patients at our clinic. We used auscultatory method as reference technique.

### Study population

Our cross–sectional study included 71 patients, treated in Cardiology Department in Vilnius University Hospital Santariskiu Klinikos since June 2014 to March 2015. Inclusion criteria were: confirmed diagnosis of nonvalvular AF and arterial hypertension. Exclusion criteria were: age less than 18 years, valvular heart disease, coronary artery disease, cerebrovascular disease, severe heart failure (New York Heart Association (NYHA) class ≥ III), hemodynamic instability, marked peripheral artery disease, clinically significant thyroid disease. AF types were classified according to the ESC guidelines. All patients were diagnosed with both AF and AH. Patients with SR and first time diagnosed AF were patients, which had AF diagnosed for the first time during this hospitalization and it was treated by medical or electrical cardioversion on the same day or few days before the BP measurement. BP was measured after the procedure, thus patients were in SR. All patients were allocated into 2 groups according to the heart rhythm at the time of BP measuring: one group consisted of patients with AF and another group included the ones in SR. The rhythm was determined by monitoring ECG before the BP measurement.

### BP measurements

Auscultatory and oscillometric BP measurements were performed using adult cuff of adequate size which was placed around the arm at heart level, with its lower edge 2–3 cm above the brachial artery pulsation point, with the patient lying in a supine position. In order to avoid venous congestion and to minimize variability in BP, the time between measurements was determined to be in a range of 1–5 min.

BP was measured according to international guidelines [9], a standard auscultatory method was used as a reference technique, as stated in established validation protocols [19, 20]. After 5 min of rest four auscultatory BP measurements were performed on the non–dominant arm. After using the auscultatory method, four oscillometric BP measurements were obtained, using a Microlife (BP A6 PC with AF detection system) device according to the manufacturer’s instructions, again with the patient in a lying position and using the same arm. Thus, an overall number of eight measurements were taken for each patient. The mean systolic and diastolic BP of these two different measurements was calculated for comparison. In this study we have also analysed the BP control. The target/controlled BP value was 140/90 mmHg or less.

The study was approved by the Local Ethical Committee of Vilnius University Hospital Santariskiu Klinikos, Vilnius, Lithuania on 10^th^ of April, 2014, protocol number EK–19.

### Statistical analysis

Results are presented as mean ± standard deviation (SD). BP values are the mean of 4 consecutive measurements. Comparison of numeric BP values of patients with and without AF was performed by paired 2-tailed *t*-tests with the 2-tailed significance level set at *p* = 0.05. BP measurement methods were compared using linear regression analysis taking auscultatory method as a reference value. Pearson’s correlation analysis was performed prior linear regression analysis. The agreement between two methods was analysed by Bland–Altman method. The assumptions of normality of differences and other characteristics were checked with a graphical approach. The resulting graph is a scatter plot in which the difference of the two paired measurements is plotted against the mean of the two measurements. Statistical analysis was performed using SPSS version 17.0.

## Results

Study included 71 patients: 36 males (mean age 67.4 years) and 35 females (70.2 years), suffering from AF and AH. The mean age of all patients was 68.8 (±9.1) years, ranging from 51 to 89 years. At the time of the BP measurement procedure, 36 (50.7%) patients were in SR and 35 (49.3%) patients had AF. Patients of both groups did not differ significantly in terms of age, sex, body mass index (BMI) and rate of smokers. The detailed characterization of study population is provided in Table 1. Antihypertensive treatment for patients with AF and SR was similar. The only difference was that more patients with SR used ACFI, while more patients with AF were on CCB, thiazide or loop diuretics and more patients with AF were on combined medication (2 or 3 drugs in one tablet). The difference was not statistically significant.

**Table 1:** Characteristics of the Study Population

Variables	AF (n, %)	SR (n, %)
Number of patients	35 (49.3)	36 (50.7)
Age (years), mean ± SD	67.5 ± 8.8	69.9 ± 9.5
Male	18 (51.4)	18 (50)
Mean value of heart rate^a^, bpm ± SD	79 ± 12.8	62 ± 6.3
BMI, mean ± SD	29.8 ± 4.8	29.7 ± 4.6
Smokers	9 (25.7)	16 (44.4)
Years of AF, mean ± SD	9.1 ± 7.9	11.9 ± 9.5
AF type:		
First time diagnosed	2 (5.7)	2 (5.6)
Paroxysmal	4 (11.4)	8 (22.2)
Persistent	12 (34.3)	26 (72.2)
Permanent	17 (48.6)	0 (0)
Blood pressure correction:		
BP <140/90 mmHg	24 (68.6)	23 (63.9)
BP >140/90 mmHg	11 (31.4)	13 (36.1)
Antihypertensive treatment:		
ACEIs/ARBs	22 (62.9)	29 (80.6)
Beta–blockers	24 (68.6)	26 (72.2)
CCBs	14 (40)	17 (47.2)
Diuretics	22 (62.9)	17 (47.2)

*AF* atrial fibrillation, *SR* sinus rhythm, *SD* standard deviation, *BMI* body mass index, *BP* blood pressure, *ACEIs* angiotensin–converting enzyme inhibitors, *ARBs* angiotensin II receptor blockers, *CCBs* calcium-channel blockers

^a^ – Mean value of heart rate, counted of 4 consecutive measurements

Data obtained by two methods of BP measuring (i.e. auscultatory method and automated oscillometry) were compared. It was found that in both groups systolic and diastolic blood pressure measurements were higher using oscillometric method (*p* = 0.007 in SR group and *p* < 0.001 in AF group for systolic BP, *p* = 0.151 in SR group and *p* = 0.032 in AF group for diastolic BP) (Table 2). In SR patients’ group mean systolic BP was 132 ± 17.9 mmHg measured by auscultatory method and 137.4 ± 19.4 mmHg measured with oscillometric device. Mean diastolic BP was 77.1 ± 10.9 mmHg (auscultatory method) and 78.5 ± 12.2 mmHg (oscillometric method). In patients with AF mean systolic BP was 127.5 ± 15.1 mmHg (auscultatory method) and 133.6 ± 17.4 mmHg (oscillometric method), mean diastolic BP was 81.4 ± 9.9 mmHg (auscultatory method) and 83.5 ± 11.8 mmHg (oscillometric method).

**Table 2:** Summary statistics of mean^a^ blood pressure by different measurements

Groups	Systolic BP by auscultatory method	Diastolic BP by auscultatory method	Systolic BP by oscillometric method	Diastolic BP by oscillometric method
Sinus rhythm	132.1 ± 17.9	77.1 ± 10.9	137.4 ± 19.4	78.5 ± 12.2
Atrial fibrillation	127.5 ± 15.1	81.4 ± 9.9	133.6 ± 17.4	83.5 ± 11.9
All	129.8 ± 16.7	79.2 ± 10.6	135.5 ± 18.4	80.9 ± 12.2

*BP* blood pressure; ^a^Mean blood pressure (mmHg) of 4 measurements by two methods ± SD

The difference between mean diastolic blood pressure, measured by auscultative and oscillometric methods did not differ significantly (*p* = 0.72), and the difference between systolic BP was lower in patients with AF (*p* = 0.19). Patients with controlled BP had a higher difference between systolic BP values measured by two methods, compared to patients with not controlled BP, *p* < 0.05.

**Fig. 1:**
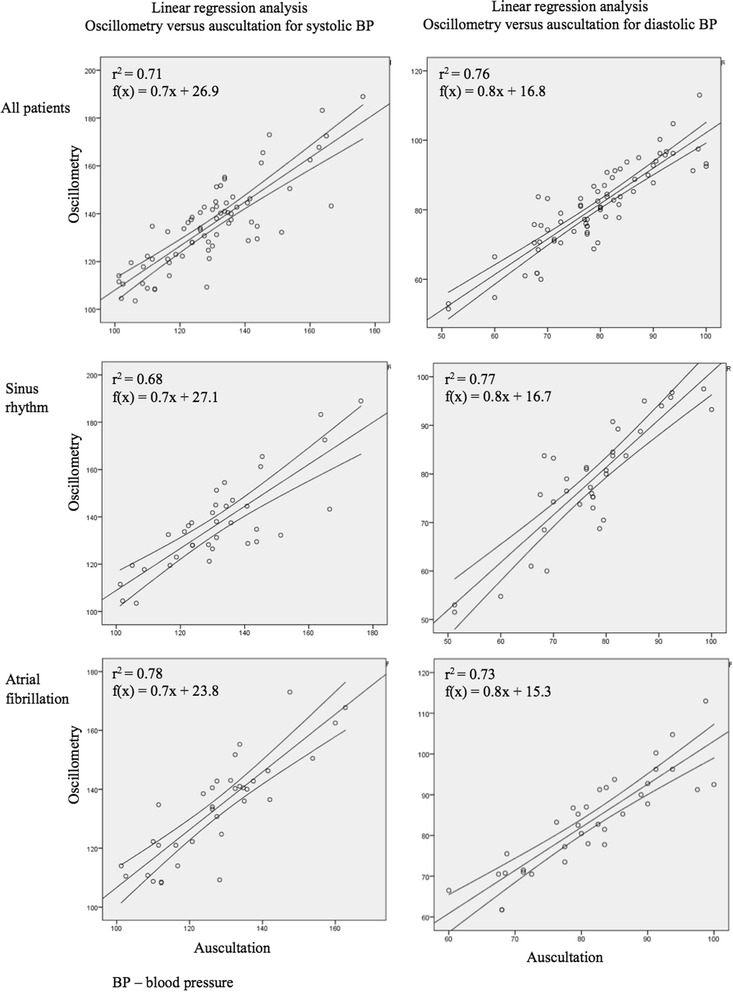
Linear regression analysis of oscillometry versus auscultation. Single linear regression models for comparison of oscillometric method versus auscultation in different patient groups (All patients, Sinus rhythm and Atrial fibrillation). The reference value is blood pressure values measured by auscultation. Results are divided by systolic and diastolic blood pressure (BP). Linear regression line is presented within 95% confidence interval

Strong correlations were observed in all investigated pairs (r value range: 0.82 – 0.88) by Pearson’s correlation analysis. regression model plots reveal that automated oscillometric BP measuring method and auscultatory method are in linear association (r^2^ = 0.71 (95%CI:0.59-0.82) for systolic BP and r^2^ = 0.76 (95%CI:0.67-0.85) for diastolic BP) (Fig. 1).

**Fig. 2:**
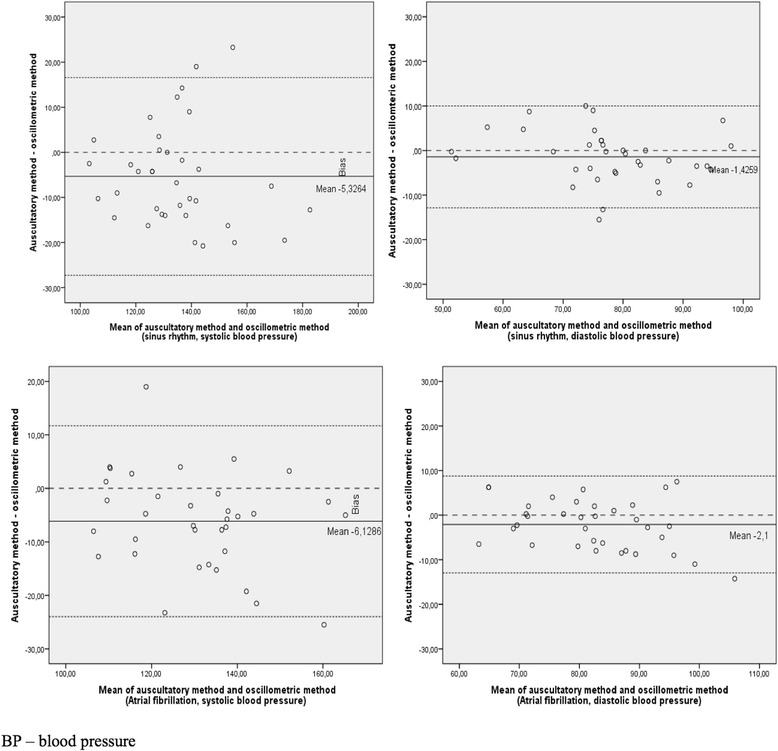
Bland–Altman analysis: plot of differences between auscultatory and oscillometric blood pressure measuring methods versus the mean of these two methods. Horizontal line represents mean difference within limits of agreement, which is defined as the mean difference ± 2 standard deviations

The mean auscultatory and oscillometry BP readings are plotted against the difference between these readings in AF and SR groups by using the Bland-Altman scatter plot format (Fig. 2). The averages of differences for SBP and DBP in SR group were (−5.3 mmHg (95% limits of agreement −27.2 – 16.6)) and (−1.4 mmHg (95% limits of agreement −12.8 – 10.0)), respectively. While for patients with AF the averages of differences for SBP and DBP were (−6.1 mmHg (95% limits of agreement −23.9 – 11.7)) and (−2.1 mmHg (95% limits of agreement −12.9 – 8.7)), respectively.

## Discussion

In this study we analysed the reliability of automated oscillometric BP measuring in hypertensive patients with AF and SR, using auscultatory method as a reference technique. In our sample all patients suffered from AH. It is known that AH has a higher prevalence in elderly and the prevalence of coincidence of AF and AH increases with age, however, it may be difficult for elderly patients to measure their BP by auscultatory method because of hearing loss or difficulties to fit the cuff properly [14].

For sustainable measurements in clinical practice, sitting position is recommended and used. We think, however, that whilst body position may influence absolute numbers and lessen the difference between systolic and diastolic blood pressures, the differences between results of measuring by different methods and their reproducibility as evaluated in this study, does not depend on body position. Many BP measurement studies have used supine position. We chose supine position because the group with atrial fibrillation was mainly inpatient subjects that were studied before scheduled electrical cardioversion, and for patients with this condition it is usually more comfortable to lie.

The results of our study are controversial comparing with recent meta-analysis [18], which analysed 566 patients with AF and showed that automated BP findings were higher than manual. Our study, as the meta-analysis, showed that BP measured with oscillometric device was higher in both AF and SR groups. However, the meta-analysis concluded that automated BP monitors in AF appear to be accurate in measuring systolic, but not diastolic BP [18], which is the opposite from our study results.

Recent cross-sectional study by Hurley and al. [21] showed that in elderly patients with AF substantially higher diastolic BP was usual, compared to the general population, regardless the BP measuring method. However, results of our study demonstrated that diastolic BP, measured with oscillometric device, is even higher than measured using auscultatory method in patients with AF.

In this study several statistical tests were used to compare two different BP measurement methods. Correlation and single linear regression analysis revealed satisfactory linear relationships between oscillometry and auscultation. However, this data does not automatically imply that there is good agreement between these two methods [22]. In order to investigate the degree of agreement, we applied the Bland–Altman analysis which is known, as one of the way for assessing compliance between two different methods of clinical measurement [23]. The results revealed that oscillometric method shows higher negative bias while measuring systolic BP in both (SR and AF) groups to compare with diastolic BP (−5.3 mmHg and −6.1 mmHg versus −1.4 mmHg and −2.1 mmHg, respectively). No significant difference in bias between SR and AF group was observed. On average the oscillometry measured BP (SBP/DBP) was 5.3/1.4 mmHg higher for patients in SR and 6.1/2.1 mmHg higher for patients with AF. Despite the fact that values of mean differences are low in our data, the “limits of agreement” [23] reveal debatable random error rates of oscilometric method for systolic BP (Fig. 2, top left and bottom left panels). Thus, the systolic BP value measured by oscillometry may be 27 mmHg higher or 17 mmHg lower in SR group and 24 mmHg higher or 12 mmHg lower in AF group above the BP values measured by auscultation. This random error is a questionable topic in clinical practice, as it could possibly affect the decision in patients’ management.

Current guidelines on treatment of AH [9] recommend repeated BP measurements in patients with arrhythmia. The study conducted by Grundvold et al. [24] found that the patients with systolic BP between 130 and 139 mmHg (“high–normal”) had a 1.5-fold risk of AF and those with systolic BP over 140 mmHg had a 1.6-fold risk, compared to patients with BP below 128 mmHg. In terms of diastolic pressures, patients with diastolic BP above 80 mmHg had a relative risk of 1.79 for developing AF, compared with patients with diastolic BP < 80 mmHg. The study concluded that increased BP over time may cause a structural and electrophysiological remodeling of atria and ventricles, increased pressure in left atrium may cause atrial dilatation, which may favor the development of AF. AFFIRM trial [25] also demonstrated that the optimal BP target in patients with permanent AF could be higher than in general population [26]. Even slight differences may determine whether patient requires more vigorous BP correction, which may lead to lower rates of cardiovascular events [27] and hopefully AF. Different results obtained by auscultatory and oscillometric BP measuring methods could be taken into account while treating AH in patients with AF.

## Conclusions

Our data show that agreement between oscillometry and auscultation is independed of AF. The oscillometric device which is validated for patients with AF on average gives 5.3 mmHg higher systolic BP values for patients with SR and 6.3 mmHg higher BP values for patients with AF. However, the limits of agreement between two methods are debatable: the systolic BP value measured by oscillometry may be 27 mmHg higher or 17 mmHg lower for patients with SR and 24 mmHg higher or 12 mmHg lower above the BP values measured by auscultation for patients in AF. The wide range of random error rates is a questionable topic in clinical practice, as it could possibly affect the treatment of arterial hypertension in patients with AF.

### Study limitation

Measurements were not alternated (auscultatory/oscillometric) or randomized, but rather performed in fixed order – 4 auscultatory followed by 4 oscillometric, thus introducing a bias relating to the effect of measurement on the measured values.

## Additional file

Additional file 1: The raw data of study patients. The dataset contains demographic data (e.g. gender, age) and clinical data (e.g. past and current diseases, blood pressure measurements) of the study patients. (XLSX 21 kb)

## Abbreviations

ACEIs: Angiotensin-converting enzyme inhibitors
AF: Atrial fibrillation
AFFIRM: Atrial fibrillation follow-up investigation of rhythm management
AH: Arterial hypertension
ARBs: Angiotensin receptor blockers
BMI: Body mass index
BP: Blood pressure
CCBs: Calcium-channel blockers
DBP: Diastolic blood pressure
NYHA: New York Heart Association
SBP: Systolic blood pressure
SD: Standard deviation
SR: Sinus rhythm
